# A New Wide-Band Double-Negative Metamaterial for C- and S-Band Applications

**DOI:** 10.3390/ma8010057

**Published:** 2014-12-25

**Authors:** Md Ikbal Hossain, Mohammad Rashed Iqbal Faruque, Mohammad Tariqul Islam, Mohammad Habib Ullah

**Affiliations:** 1 Space Science Center (ANGKASA), Research Centre Building, Universiti Kebangsaan Malaysia, Bangi, Selangor D.E. 43600, Malaysia; E-Mail: rashed@ukm.edu.my; 2 Department of Electrical, Electronic and Systems Engineering, Faculty of Engineering and Built Environment, Universiti Kebangsaan Malaysia, Bangi, Selangor D.E. 43600, Malaysia; E-Mail: tariqul@ukm.edu.my; 3 Department of Electrical Engineering, Faculty of Engineering, University of Malaya, Kuala Lumpur 50603, Malaysia; E-Mail: mhullah@yahoo.com

**Keywords:** C-band, finite-difference time-domain (FDTD) method, double-negative (DNG) metamaterial, metamaterial unit-cell, metamaterial array, S-band

## Abstract

A new design and analysis of a wide-band double-negative metamaterial, considering a frequency range of 0.5 to 7 GHz, is presented in this paper. Four different unit cells with varying design parameters are analyzed to evaluate the effects of the unit-cell size on the resonance frequencies of the metamaterial. Moreover, open and interconnected 2 × 2 array structures of unit cells are analyzed. The finite-difference time-domain (FDTD) method, based on the Computer Simulation Technology (CST) Microwave Studio, is utilized in the majority of this investigation. The experimental portion of the study was performed in a semi-anechoic chamber. Good agreement is observed between the simulated and measured S parameters of the developed unit cell and array. The designed unit cell exhibits negative permittivity and permeability simultaneously at S-band (2.95 GHz to 4.00 GHz) microwave frequencies. In addition, the designed unit cell can also operate as a double-negative medium throughout the C band (4.00 GHz to 4.95 GHz and 5.00 GHz to 5.57 GHz). At a number of other frequencies, it exhibits a single negative value. The two array configurations cause a slight shift in the resonance frequencies of the metamaterial and hence lead to a slight shift of the single- and double-negative frequency ranges of the metamaterial.

## 1. Introduction

Metamaterials, or left-handed media, have been frequently discussed in recent years because they exhibit certain extraordinary electromagnetic properties at specific frequency bands. Essentially, metamaterials are artificially constructed materials that can exhibit negative permittivity and/or negative permeability [1,2]. The characteristic behavior of metamaterials can be achieved by implementing certain artificial material structures rather than by using specific chemical compositions. Metamaterials can be classified into three categories: zero-index materials, single-negative media, and double-negative media. When both the permittivity and the permeability of a material are equal to zero over a certain frequency range, then it is called a zero-index material [3,4]. A material with either a negative permittivity or a negative permeability is known as a single-negative (SNG) medium [5]. On the other hand, a material with both negative permittivity and negative permeability is called a double-negative (DNG) medium [6].

In 1968, Victor Veselago [7] introduced the first DNG media with negative ε and μ, which exhibited certain unique properties compared with ordinary material. In 2000, a DNG material was successfully demonstrated by Smith *et al.* [8] to exhibit negative ε and μ simultaneously. Because of their extraordinary features, metamaterials can be used for a wide variety of applications. Super lenses fabricated using metamaterials demonstrate resolutions three times better than those of ordinary lenses [9]. Metamaterials can also be used in biomedical applications [10] and invisibility cloaks [11]. Moreover, metamaterials are widely used in various types of antennas. Antenna performance is enhanced by the use of metamaterial [12,13]. The use of metamaterial in antennas also reduces their electromagnetic health hazards by reducing the specific absorption rate (SAR) of associated radiation in the human head [2,14]. In [15], a metamaterial absorber for terahertz frequencies was proposed. In [16], a double S-shaped metamaterial was proposed for Ku-band applications. In [1], the design and fabrication of metamaterials for X-band application were reported. The results revealed double-negative characteristics over a range of 9.2 GHz to 10.1 GHz, a frequency bandwidth of approximately 1 GHz. In [17], a metamaterial-embedded microstrip patch antenna was proposed for WLAN application. The results indicated a negative value of permeability spanning a narrow frequency band from 8 to 8.4 GHz. In the study reported in [18], a metamaterial array was used for the enhancement of antenna gain in the S band. The presented results indicated negative permittivity (ɛ) over a narrow band (2.23 GHz to 2.4 GHz). However, the size of the presented metamaterial array was not compatible with that of modern handsets. In [19], an H-shaped metamaterial was proposed for multiband microwave applications. Double left-handed characteristics were observed in the C (0.5 GHz bandwidth) and S (0.3 GHz bandwidth) bands, but the size of the unit cell would not be suitable for most microwave applications. The size of the metamaterial unit-cell design proposed in [19] is 30 × 30 mm^2^, which is quite a bit larger than that of the unit-cell design presented in this manuscript. The ratio of resonant wavelength (λ_0_) to unit-cell size (*a*) of [19] is not large enough and hence, it is not suitable to operate a sub wavelength regime. In addition, the thickness of the substrate of the unit cell in [19] is twice that used here. However, this design provides more DNG bandwidth in the C- and S-bands compared with that of [19].

In this paper, the design and analysis of a new wide-band metamaterial for microwave C- and S-band applications are presented. The proposed metamaterial exhibits negative permittivity and permeability simultaneously over bandwidths of approximately 1.05 GHz in the microwave C band and approximately 1.62 GHz in the microwave S band. Moreover, the present design is compact in size and hence low in fabrication cost. This metamaterial can be used in modern compact devices such as cellular phones, electronic devices, biomedical equipment, *etc*.

## 2. Metamaterial Construction

**Figure 1:**
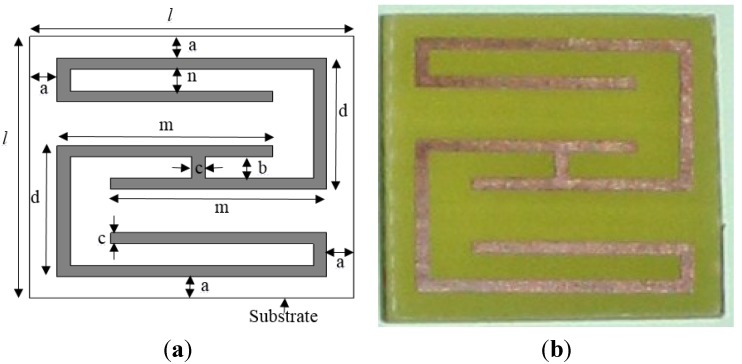
Metamaterial unit cell A: (**a**) proposed geometry; and (**b**) fabricated prototype.

The developed metamaterial structure is depicted in Figure 1a, with the substrate. The developed structure consists of two G-shaped split-square resonators connected to each other. The structure is fabricated from a copper sheet of 0.035 mm in thickness, and the substrate material is FR-4 glass epoxy. The dielectric constant and tangent loss of the substrate are 4.3 and 0.025, respectively. For the design of the metamaterial, a square-shaped substrate of 0.8 mm in thickness is used. Four different unit cells (12 × 12 mm^2^, 16 × 16 mm^2^, 20 × 20 mm^2^, and 24 × 24 mm^2^) with varying structure and substrate parameters were analyzed to evaluate the effects of the unit-cell size on the resonance frequencies of the metamaterial. All parameters of the four different unit cells are listed in Table 1, where the unit cells are labeled A, B, C, and D in order of increasing size. A fabricated prototype of unit cell A was used for measurement purposes, as indicated in Figure 1b. The structure behaves as an LC resonator circuit. In this structure, the length of the printed metal strip is responsible for the inductance, and the splits are the origin of the capacitance. Together, this inductance and capacitance determine the resonance frequencies of the material.

**Table 1:** Unit-cell design parameters.

Unit-Cell Parameters	Value (mm)			
	Unit Cell A	Unit Cell B	Unit Cell C	Unit Cell D
*a*	1	1	1	1
*b*	2	2	2	2
*c*	0.5	0.5	0.5	0.5
*d*	6	8	10	12
*l*	12	16	20	24
*m*	8	12	16	20
*n*	2.5	3	3.5	4

## 3. Numerical Methods

The reflection and transmission parameters of the metamaterial unit cells and arrays were calculated to determine the electromagnetic behaviors of the proposed structures. The Nicolson Rose Weir (NRW) method [20,21] was utilized to extract the effective relative permittivity (${\text{ε}}_{\text{r}}^{\text{eff}}$), permeability (${\text{μ}}_{\text{r}}^{\text{eff}}$) and refractive index (*n*_eff_). The retrieval of effective parameters from reflection and transmission data depends on the facts that unit-cell dimension should be much smaller than the operating wavelength in the media [22,23,24].

At the interface between the slab (thickness *d*) and free space, the reflection coefficient can be expressed as follows:

(1) $$\Gamma =\frac{Z_{0}-1}{Z_{0}+1}$$

where *Z*_0_ is the relative impedance in terms of permittivity and permeability.

(2) $$Z_{0}=\sqrt{{\text{μ}}_{\text{r}}{\text{/ε}}_{\text{r}}}$$

The S parameters S_11_ and S_21_ are expressed as follows:

(3) $$S_{21}=\frac{(1-\Gamma^{2})z}{1-\Gamma^{2}z^{2}}$$

(4) $$S_{11}=\frac{(1-z^{2})\Gamma }{1-\Gamma^{2}z^{2}}$$

where the transmission coefficient is expressed as $z=\exp(-j(w/c)\sqrt{{\text{μ}}_{\text{r}}{\text{ε}}_{\text{r}}}d$, where c is the velocity of light, and ω=2πf is the angular frequency.

Using a similar approach to that described in [1], the following equations can be written:

(5) $${\text{μ}}_{\text{r}}=\frac{2c(1-S_{21}+S_{11})}{j\text{ω}d(1+S_{21}-S_{11})}$$

(6) $${\text{ε}}_{\text{r}}={\text{μ}}_{\text{r}}+j\frac{2cS_{11}}{\text{ω}d}$$

(7) $$n_{\text{r}}=\sqrt{{\text{μ}}_{\text{r}}{\text{ε}}_{\text{r}}}$$

**Figure 2:**
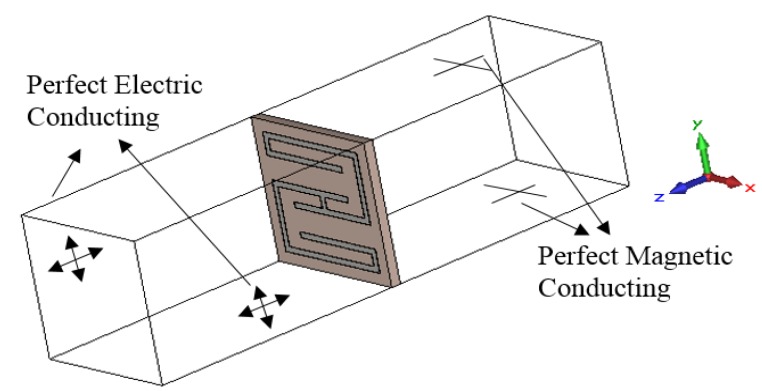
Simulation set-up for the metamaterial unit cell in the Computer Simulation Technology Microwave Studio (CST MWS).

The scattering parameters of the unit cell were calculated using the FDTD method in CST Microwave Studio (Computer Simulation Technology AG, Darmstadt, Germany). In the simulation setup, perfect electric and magnetic boundaries were imposed on the metamaterial unit cell, and the cell was placed between two waveguide ports for testing. Figure 2 illustrates the orientation of the metamaterial unit cell in the CST MWS Studio simulation setup. The perfectly electrically conducting and perfectly magnetically conducting boundary conditions were defined in the *x* and *y* directions in the simulation setup, and the structure was excited by a uniform plane wave propagating in the *z* direction. The frequency range of 0.5 GHz to 7 GHz was considered by the frequency-domain solver for the simulation of the metamaterial structure. A tetrahedral mesh with the adaptive mesh scheme was utilized in this investigation. Moreover, the open and interconnected array is tested using similar boundaries and mesh setting as a unit cell.

## 4. Results and Discussion

In this paper, the electromagnetic behavior of the proposed metamaterial is explained using the real values of the effective permittivity (${\text{ε}}_{\text{r}}^{\text{eff}}$), effective permeability (${\text{μ}}_{\text{r}}^{\text{eff}}$), and refractive index (*n*_eff_) for the unit cells and arrays. In addition, the transmission parameter S_21_ for each unit cells is presented to clarify the characteristics of the metamaterial.

### 4.1. Unit Cells

**Figure 3:**
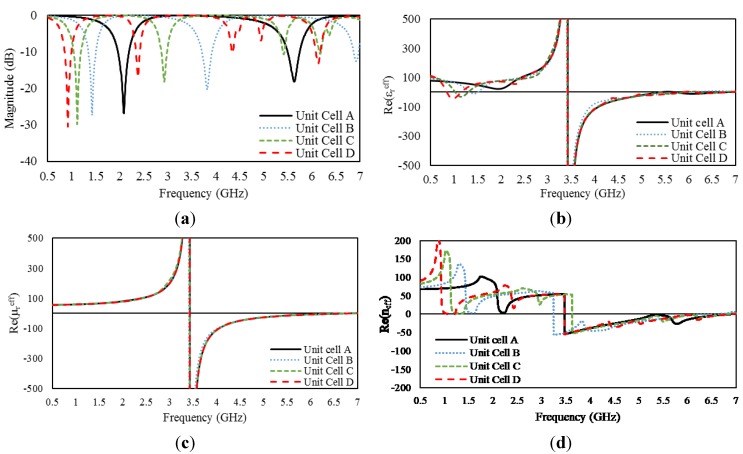
(**a**) S_21_ curves for the different unit cells; (**b**) real part of the effective permittivity; (**c**) real part of the effective permeability; and (**d**) real part of the refractive index.

Figure 3a presents the S_21_ curves for the four different unit cells from 0.5 GHz to 7 GHz. The unit cells have similar structures but different sizes. The results reveal a significant variation in the resonant frequencies caused by the variation in unit-cell size. Unit cell A (12 × 12 mm^2^) produces resonance at 2.1 and 5.6 GHz. For unit cell B (16 × 16 mm^2^), these resonance frequencies are shifted to 1.4 and 3.8 GHz, respectively. For a further increase in size, unit cell C (20 × 20 mm^2^) exhibits three resonances at 1.12, 2.94 and 5.43 GHz. Similarly, unit cell D (24 × 24 mm^2^) exhibits resonances at frequencies of 0.92, 2.4, 4.4, and 5.02 GHz. As the size of the structure increases, the resonance frequencies shift toward lower frequencies and the number of resonances increases. It is also observed that the resonances at higher frequencies are not as strong as those at lower frequencies for all unit cells.

The real parts of the effective permittivities and permeabilities of the unit cells are plotted in Figure 3b,c, respectively. The different unit cells exhibit slight variation in metamaterial characteristics. Unit cell A exhibits negative ${\text{ε}}_{\text{r}}^{\text{eff}}$ values for frequencies from 2.95 to 4.96 GHz and from 5.67 to 6 GHz and negative ${\text{μ}}_{\text{r}}^{\text{eff}}$ values from 2.95 to 5.82 GHz. Thus, unit cell A behaves as a double-negative medium over frequency ranges of 2.95 to 4.96 GHz and 5.67 to 5.82 GHz and as a single-negative metamaterial from 4.96 to 5.67 GHz and from 5.82 to 6 GHz. Unit cell B exhibits double-negative characteristics from 2.95 to 5.53 GHz and single-negative characteristics from 1.37 to 1.4 GHz and from 5.53 to 5.89 GHz. Similarly, unit cell C exhibits two negative values over ranges from 2.92 to 5.1 GHz and from 5.45 to 5.75 GHz and a single negative value over ranges from 1.04 to 1.25 GHz, from 5.1 to 5.45 GHz, and from 5.75 to 5.88 GHz. Finally, unit cell D behaves as a single-negative metamaterial from 0.85 to 1.09 GHz, from 4.96 to 5.03 GHz, and from 5.57 to 5.88 GHz and as a double-negative metamaterial at frequencies from 2.95 to 4.96 GHz and from 5.03 to 5.57 GHz. Figure 3d presents the real parts of the effective refractive indices of the different unit cells. The presented results clearly demonstrate that unit cells of larger size behave as metamaterials over broader ranges of frequency.

**Figure 4:**
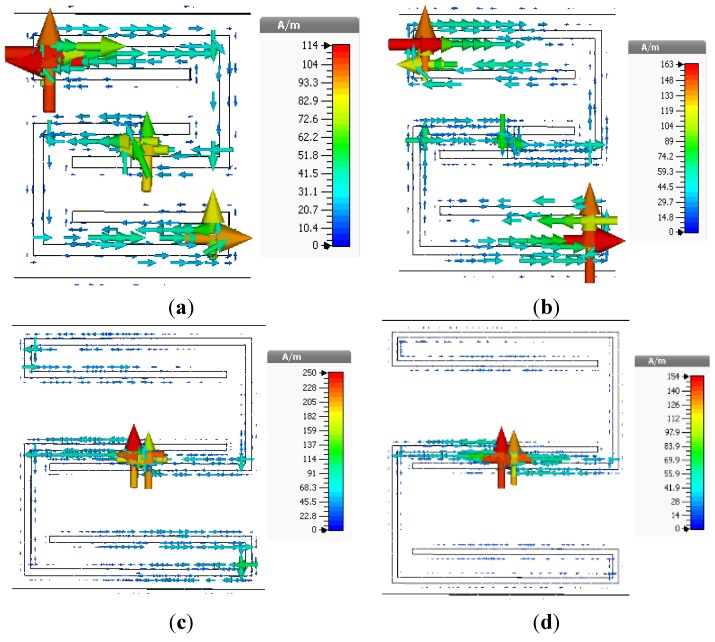
Surface current distributions of unit cells at 3.5 GHz, (**a**) unit cell A; (**b**) unit cell B; (**c**) unit cell C; and (**d**) unit cell D.

Figure 4 depicts the surface current distributions of the unit cells at 3.5 GHz, where all unit cells exhibit double-negative characteristics. For unit cells of different sizes, the maximum surface current occurs at different phases. For unit cell A, the maximum surface current is 114 A/m at a phase of 22°. Unit cell B produces a maximum surface current of 163 A/m at a phase of 67°. Similarly, the maximum surface current for unit cell C is 250 A/m at a phase of 18°, and that for unit cell D is 154 A/m at a phase of 30°. In the negative-permeability frequency range of a unit cell, the current begins to lag with respect to the applied field.

### 4.2. Arrays

**Figure 5:**
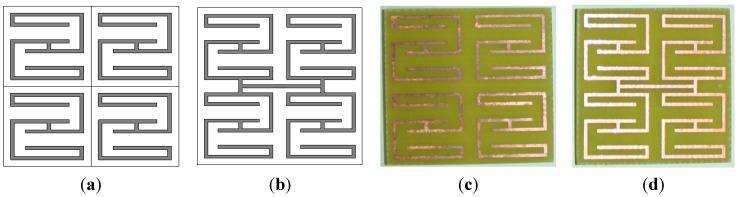
2 × 2 array configuration of unit cells: (**a**) open array structure; (**b**) interconnected array structure; (**c**) prototype of an open array; and (**c**) prototype of an interconnected array.

Two types of array configurations are investigated in this section for all unit cells. First, an array configuration called the open array configuration, in which the unit cells are not connected to each other, as illustrated in Figure 5a, is considered. Second, an array of interconnected unit cells, called the interconnected array configuration and depicted in Figure 5b, is considered. Fabricated prototypes of open and interconnected 2 × 2 arrays of unit cell A are shown in Figure 5c,d, respectively. The effective parameters of the arrays are presented for a frequency range of 0.5 GHz to 6 GHz.

#### 4.2.1. 2 × 2 Arrays of Unit Cell A

**Figure 6:**
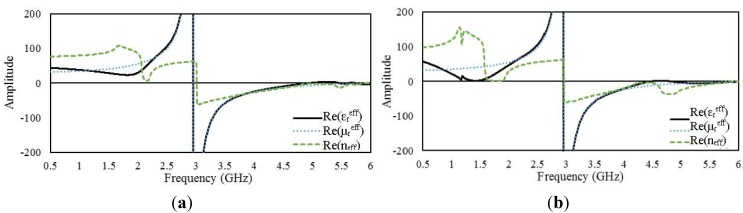
Real parts of the effective parameters of 2 × 2 arrays of unit cell A: (**a**) open array and (**b**) interconnected array.

**Figure 7:**
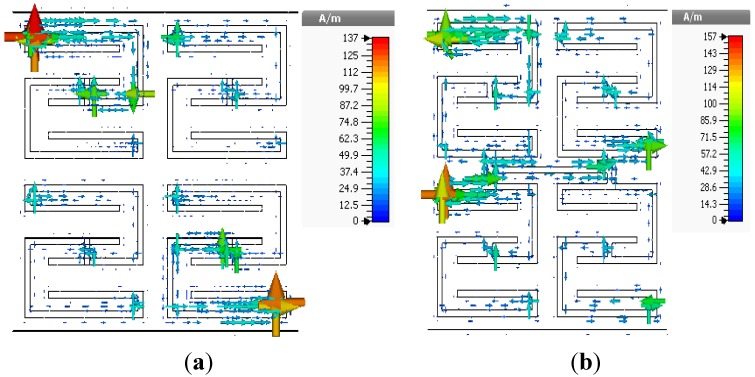
Surface current distributions of 2 × 2 arrays of unit cell A at 3.5 GHz: (**a**) open array and (**b**) interconnected array.

Figure 6a,b presents the real parts of the effective parameters of the open and interconnected arrays, respectively, of unit cell A. The open and interconnected arrays exhibit slight shifts of their resonance points with respect to the unit-cell characteristics. The open array exhibits double negative values from 2.95 to 4.93 GHz and from 5.75 to 5.83 GHz, and the interconnected array exhibits this behavior from 2.95 to 4.47 GHz and from 4.83 to 5.89 GHz. In addition, the interconnected array provides near-zero ${\text{ε}}_{\text{r}}^{\text{eff}}$ values over a frequency range of 1.3 to 1.5 GHz. The surface current distributions of the open and interconnected arrays at the same frequency are presented in Figure 7. The interconnected array yields a slightly higher surface current with some phase difference.

#### 4.2.2. 2 × 2 Arrays of Unit Cell B

**Figure 8:**
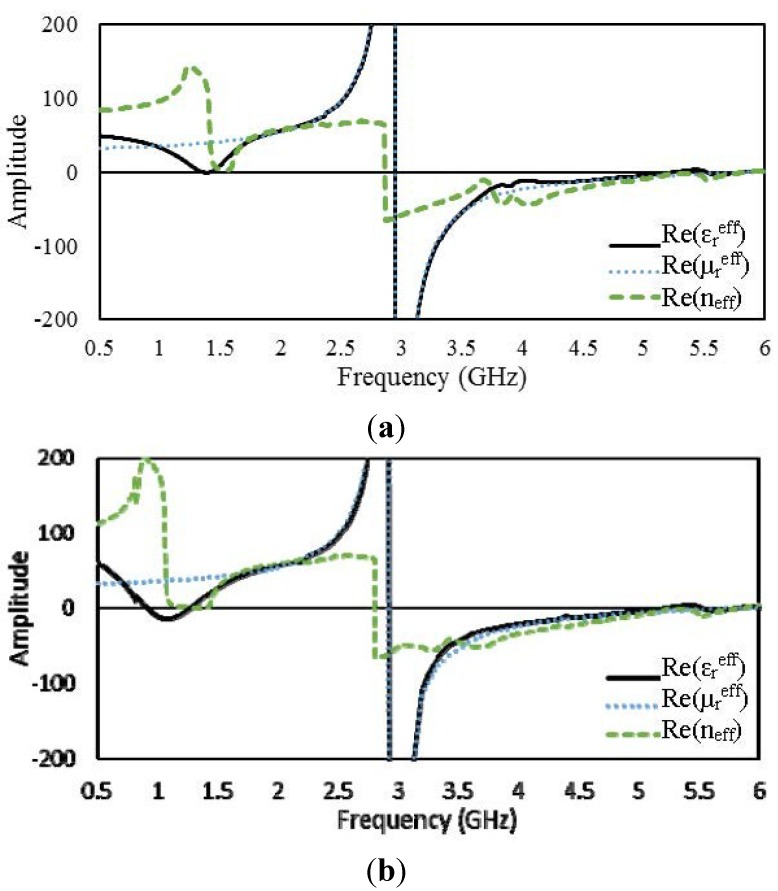
Real parts of the effective parameters of 2 × 2 arrays of unit cell B: (**a**) open array and (**b**) interconnected array.

**Figure 9:**
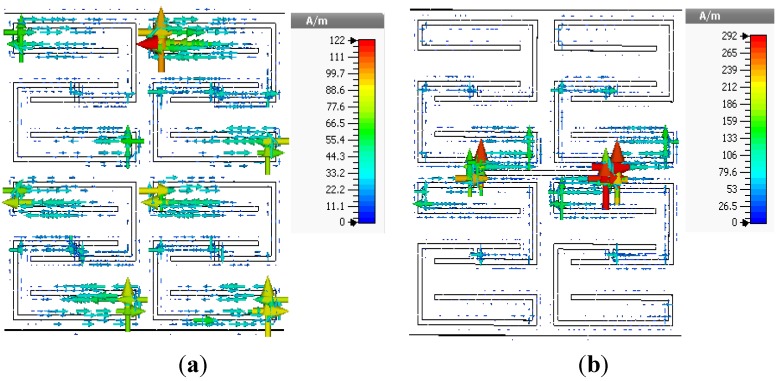
Surface current distributions of 2 2 × 2 array of unit cell B at 3.5 GHz: (**a**) open array and (**b**) interconnected array.

Figure 8 presents the real parts of the effective parameters of the open and interconnected arrays of unit cell B. The open array exhibits negative ${\text{ε}}_{\text{r}}^{\text{eff}}$ over three different frequency ranges: from 1.3 to 1.42 GHz, from 2.95 to 5.24 GHz, and from 5.52 to 5.8 GHz. On the other hand, for the interconnected array, a shift is observed in the lowest-frequency resonance band; the interconnected array exhibits negative ${\text{ε}}_{\text{r}}^{\text{eff}}$ values from 0.9 to 1.27 GHz. For the other two frequency ranges, the interconnected array exhibits similar values to those of the open array. Moreover, both arrays exhibit negative ${\text{μ}}_{\text{r}}^{\text{eff}}$ values from 2.95 to 2.9 GHz. Figure 9 presents the surface current distributions of the open and interconnected arrays of unit cell B at 3.5 GHz. The surface current density is more significant in the interconnection region for the interconnected array.

#### 4.2.3. 2 × 2 Arrays of Unit Cell C

**Figure 10:**
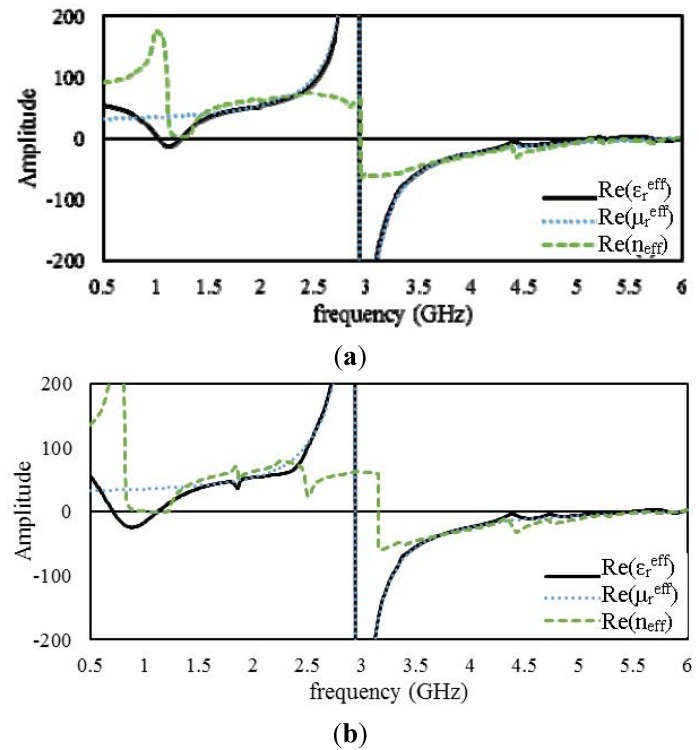
Real parts of the effective parameters of 2 × 2 arrays of unit cell C: (**a**) open array and (**b**) interconnected array.

**Figure 11:**
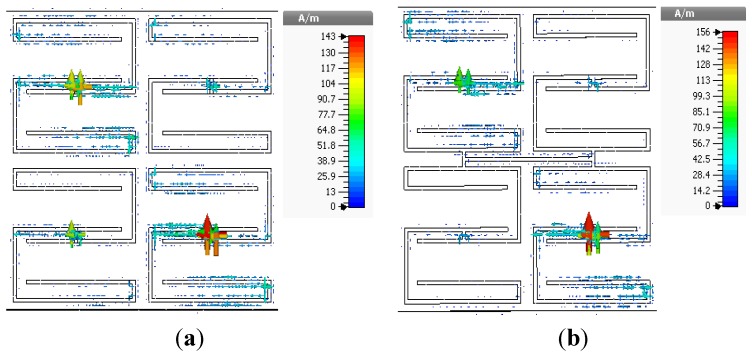
Surface current distributions of 2 × 2 arrays of unit cell C at 3.5 GHz: (**a**) open array and (**b**) interconnected array.

Figure 10 presents the real parts of the effective parameters for arrays of unit cell C. As in the case of the arrays of unit cells A and B, the open array of unit cell C exhibits characteristics very similar to those of the corresponding unit cell, and the differences observed for the interconnected array primarily affect the ${\text{ε}}_{\text{r}}^{\text{eff}}$ values in the lower-frequency range. The open array exhibits double negative values from 2.94 to 5.12 GHz and from 5.7 to 5.87 GHz, and the interconnected array exhibits double-negative behavior from 2.95 to 5.46 GHz and from 5.82 to 5.85 GHz. The results indicate slight frequency shifts of both the double-negative and single-negative regions for the interconnected array configuration. The surface current distributions of the open and interconnected arrays are presented in Figure 11 The maximum values of the surface current for the open and interconnected arrays are 143 A/m and 156 A/m, respectively.

#### 4.2.4. 2 × 2 Arrays of Unit Cell D

**Figure 12:**
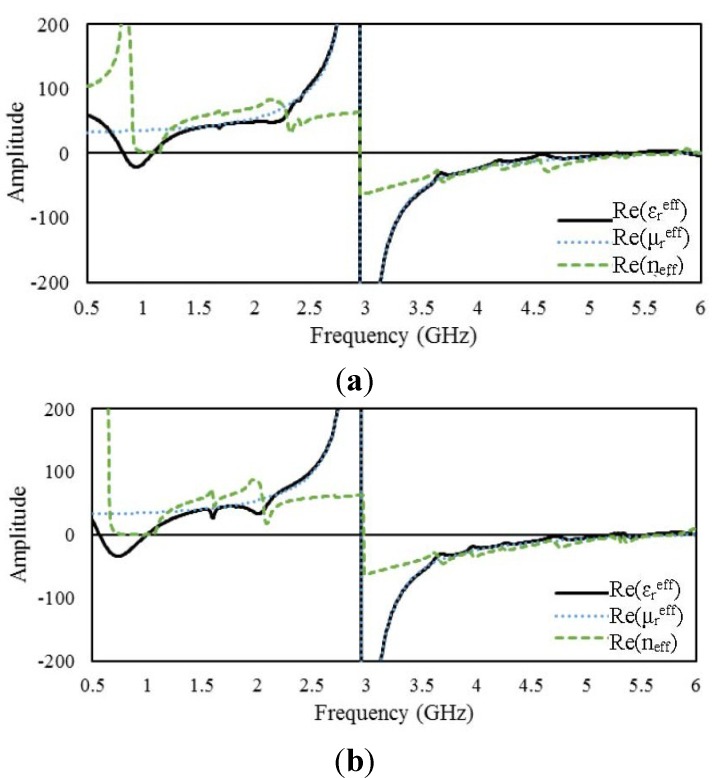
Real parts of the effective parameters of 2 × 2 arrays of unit cell D: (**a**) open array and (**b**) interconnected array.

**Figure 13:**
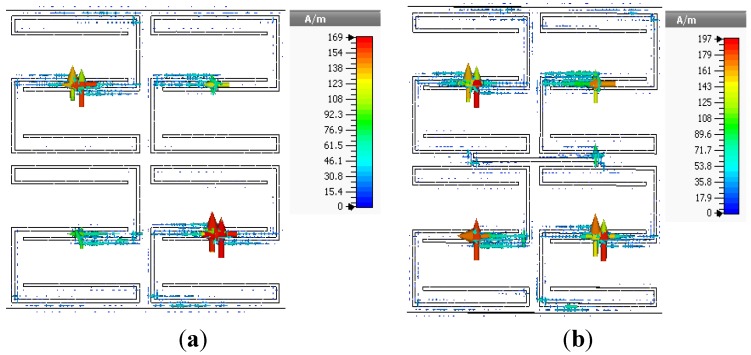
Surface current distributions of 2 × 2 array of unit cell D at 3.5 GHz: (**a**) open array and (**b**) interconnected array.

Figure 12 presents the real parts of ${\text{ε}}_{\text{r}}^{\text{eff}}$, ${\text{μ}}_{\text{r}}^{\text{eff}}$ and *n*_eff_ for the open and interconnected arrays of unit cell D. For the open array, ${\text{ε}}_{\text{r}}^{\text{eff}}$ becomes negative over frequency ranges from 0.82 to 1.09 GHz, from 2.95 to 5.21 GHz, from 5.26 to 5.4 GHz, and from 5.88 to 6 GHz. On the other hand, the ranges in which the interconnected array exhibits negative ${\text{ε}}_{\text{r}}^{\text{eff}}$ values are shifted to lower frequencies: from 0.57 to 0.99 GHz, from 2.95 to 5.21 GHz, and from 5.36 to 5.55 GHz. Both arrays exhibit negative permeability in the same frequency range. Figure 13 presents the surface current distributions of the open and interconnected arrays of unit cell D.

## 5. Experimental Validation

**Figure 14:**
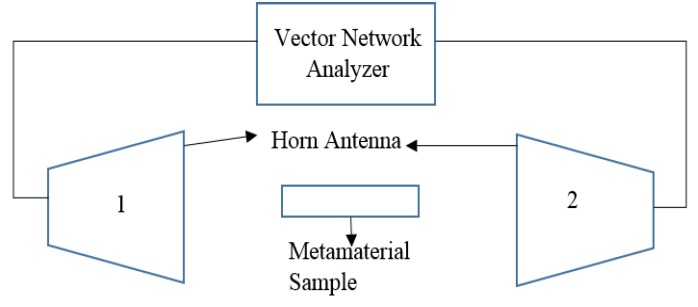
Experimental set-up for the measurement of S parameters.

A prototype of unit cell A was fabricated for measurement to validate the simulation results. Open and interconnected 2 × 2 arrays of unit cell A were also fabricated. The experiments were performed in a semi-anechoic chamber using two broadband horn antennas placed 1.5 m apart. The prototypes were placed between the horn antennas in the same plane, analogous to the simulation geometry. An Agilent E8363D vector network analyzer was utilized to determine the transmission parameters. The experimental set-up in the anechoic chamber is depicted in Figure 14.

**Figure 15:**
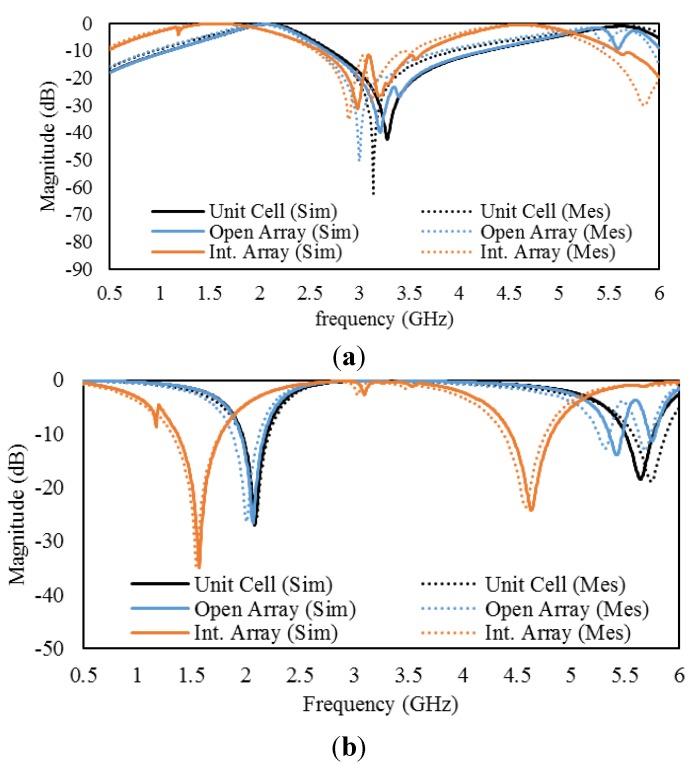
S parameters for unit cell A and arrays thereof: (**a**) reflection parameters (S_11_) and (**b**) transmission parameters (S_21_).

The simulated and measured S parameters (S_11_ and S_21_) of the unit cell A and the arrays thereof are presented in Figure 15. The results reveal that the measured transmission (S_21_) and reflection (S_11_) parameters of the unit-cell and the arrays agree well with the corresponding simulations. For the unit cell and the arrays, the measured S_11_ value exhibits a slight shift in the resonances toward lower frequencies than those indicated by the simulation. This shift can most likely be attributed to fabrication error and connector issues. For the transmission parameter spectra of the unit cell and the arrays, no noticeable frequency shifts were observed in the measured S_21_ resonances compared with the simulations.

## 6. Conclusions

In this paper, a new design for a metamaterial unit-cell structure and corresponding array configurations are presented. The effective parameters and transmission coefficients of the proposed metamaterial were analyzed over a frequency range from 0.5 to 7 GHz. The results reveal double- and single-negative metamaterial characteristics of the unit cells and arrays in multiple frequency bands throughout this range. The designed unit cell exhibits double-negative characteristics at S-band and C-band microwave frequencies. The results also indicate that for unit cells of larger size, the negative metamaterial characteristics shift toward lower frequencies. The interconnected array configuration allows for a higher surface current by virtue of the interconnection of the unit cells and leads to a further shift of the resonance frequencies to lower-frequency ranges. Moreover, the sizes and transmission coefficients of the proposed metamaterial unit cells and arrays are sufficiently small for implementation in modern radio, satellite, and cellular communication applications.

## References

[1] Ziolkowski R.W. (2003). Design, fabrication, and testing of double negative metamaterials. IEEE Trans. Antennas Propag..

[2] Hossain M.I., Mohammad R.I.F., Islam M.T., Hanafi N.H.M. (2014). Application of auxiliary antenna elements for SAR reduction in the human head. Advanced Materials Research.

[3] Huang X.Q., Lai Y., Hang Z.H., Chan C.T. (2011). Dirac cones induced by accidental degeneracy in photonic crystal and zero-refractive-index materials. Nat. Mater..

[4] Ziolkowski R.W. (2004). Propagation in and scattering from a matched metamaterial having a zero index of refraction. Phys. Rev. E.

[5] Cui T.J., Smith D., Liu R. (2009). Metamaterials: Theory, Design, and Applications.

[6] Karamanos T.D., Dimitriadis A.I., Kantartzis N.V. (2012). Compact double-negative metamaterials based on electric and magnetic resonators. Antennas Wirel. Propag. Lett. IEEE.

[7] Viktor G., Veselago P.N.L. (1968). The electrodynamics of substances with simultaneously negative values of ɛ and μ. Sov. Phys. Uspekhi.

[8] Smith D.R., Padilla W.J., Vier D.C., Nemat-Nasser S.C., Schultz S. (2000). Composite medium with simultaneously negative permeability and permittivity. Phys. Rev. Lett..

[9] Grbic A., Eleftheriades G.V. (2004). Overcoming the diffraction limit with a planar left-handed transmission-line lens. Phys. Rev. Lett..

[10] Puentes M., Maasch M., Schussler M., Jakoby R. (2012). Frequency multiplexed 2-dimensional sensor array based on split-ring resonators for organic tissue analysis. IEEE Trans. Microw. Theory Tech..

[11] Schurig D., Mock J.J., Justice B.J., Cummer S.A., Pendry J.B., Starr A.F., Smith D.R. (2006). Metamaterial electromagnetic cloak at microwave frequencies. Science.

[12] Ullah M.H., Islam M.T., Faruque M.R.I. (2013). A near-zero refractive index meta-surface structure for antenna performance improvement. Materials.

[13] Wu B.-I., Wang W., Pacheco J., Chen X., Grzegorczyk T.M., Kong J.A. (2005). A study of using metamaterials as antenna substrate to enhance gain. Prog. Electromagn. Res..

[14] Faruque M.R.I., Islam M.T., Ali M.A.M. (2013). A new design of metamaterials for SAR reduction. Meas. Sci. Rev..

[15] Landy N.I., Bingham C.M., Tyler T., Jokerst N., Smith D.R., Padilla W.J. (2009). Design, theory, and measurement of a polarization-insensitive absorber for terahertz imaging. Phys. Rev. B.

[16] Chen H., Ran L., Huangfu J., Zhang X., Chen K., Grzegorczyk T.M., Kong J.A. (2004). Left-handed materials composed of only S-shaped resonators. Phys. Rev. E.

[17] Joshi J.G., Pattnaik S.S., Devi S. (2012). Metamaterial embedded wearable rectangular microstrip patch antenna. Int. J. Antennas Propag..

[18] Attia H., Bait-Suwailam M.M., Ramahi O.M., Electromagnet A. (2010). Enhanced gain planar inverted-F antenna with metamaterial superstrate for UMTS applications. PIERS Online.

[19] Islam S.S., Faruque M.R.I., Islam M.T. (2014). The design and analysis of a novel Split-H-Shaped metamaterial for multi-band microwave applications. Materials.

[20] Nicolson A.M., Ross G.F. (1970). Measurement of the intrinsic properties of materials by time-domain techniques. IEEE Trans. Instrum. Meas..

[21] Barroso J.J., de Paula A.L. (2010). Retrieval of permittivity and permeability of homogeneous materials from scattering parameters. J. Electromagn. Waves Appl..

[22] Schurig D., Mock J.J., Smith D.R. (2006). Electric-field-coupled resonators for negative permittivity metamaterials. Appl. Phys. Lett..

[23] Liu R., Degiron A., Mock J.J., Smith D.R. (2007). Negative index material composed of electric and magnetic resonators. Appl. Phys. Lett..

[24] Li D., Szabo Z., Qing X., Li E.-P., Chen Z.N. (2012). A high gain antenna with an optimized metamaterial inspired superstrate. IEEE Trans. Antennas Propag..

